# A genetic map of cassava (*Manihot esculenta* Crantz) with integrated physical mapping of immunity-related genes

**DOI:** 10.1186/s12864-015-1397-4

**Published:** 2015-03-16

**Authors:** Johana Carolina Soto, Juan Felipe Ortiz, Laura Perlaza-Jiménez, Andrea Ximena Vásquez, Luis Augusto Becerra Lopez-Lavalle, Boby Mathew, Jens Léon, Adriana Jimena Bernal, Agim Ballvora, Camilo Ernesto López

**Affiliations:** Manihot Biotec Laboratory, Biology Department, Universidad Nacional de Colombia, Bogotá, Colombia; Laboratory of Mycology and Plant Pathology, Universidad de los Andes, Bogotá, Colombia; International Center for Tropical Agriculture (CIAT), Cali, Colombia; INRES-Plant Breeding University of Bonn, Bonn, Germany; Present address Department of Biological Sciences, Vanderbilt University, Nashville, TN USA; Present address Max Planck Institute for Molecular Plant Physiology, Potsdam-Golm, Germany

**Keywords:** Linkage mapping, Physical mapping, Genotyping by sequencing, Single nucleotide polymorphisms, Immunity-related genes

## Abstract

**Background:**

Cassava, *Manihot esculenta* Crantz, is one of the most important crops world-wide representing the staple security for more than one billion of people. The development of dense genetic and physical maps, as the basis for implementing genetic and molecular approaches to accelerate the rate of genetic gains in breeding program represents a significant challenge. A reference genome sequence for cassava has been made recently available and community efforts are underway for improving its quality. Cassava is threatened by several pathogens, but the mechanisms of defense are far from being understood. Besides, there has been a lack of information about the number of genes related to immunity as well as their distribution and genomic organization in the cassava genome.

**Results:**

A high dense genetic map of cassava containing 2,141 SNPs has been constructed. Eighteen linkage groups were resolved with an overall size of 2,571 cM and an average distance of 1.26 cM between markers. More than half of mapped SNPs (57.4%) are located in coding sequences. Physical mapping of scaffolds of cassava whole genome sequence draft using the mapped markers as anchors resulted in the orientation of 687 scaffolds covering 45.6% of the genome. One hundred eighty nine new scaffolds are anchored to the genetic cassava map leading to an extension of the present cassava physical map with 30.7 Mb. Comparative analysis using anchor markers showed strong co-linearity to previously reported cassava genetic and physical maps. *In silico* based searching for conserved domains allowed the annotation of a repertory of 1,061 cassava genes coding for immunity-related proteins (IRPs). Based on physical map of the corresponding sequencing scaffolds, unambiguous genetic localization was possible for 569 IRPs.

**Conclusions:**

This is the first study reported so far of an integrated high density genetic map using SNPs with integrated genetic and physical localization of newly annotated immunity related genes in cassava. These data build a solid basis for future studies to map and associate markers with single loci or quantitative trait loci for agronomical important traits. The enrichment of the physical map with novel scaffolds is in line with the efforts of the cassava genome sequencing consortium.

**Electronic supplementary material:**

The online version of this article (doi:10.1186/s12864-015-1397-4) contains supplementary material, which is available to authorized users.

## Background

The advent and progress made in the last two decades of DNA based molecular markers has contributed to the generation of dense genetic maps [1-3]. New technologies like next generation sequencing (NGS) have made possible the high throughput identification and genotyping of thousands of molecular markers in a relatively short time and potentially at a low cost [4]. A fast cost-effective approach to next-generation molecular marker discovery called genotyping by sequencing (GBS), has been proposed to reduce the turnaround time significantly and increases the availability of thousands of SNP (single nucleotide polymorphism) molecular markers evenly distributed throughout the genome [2,5].

High-density genetic maps built using SNPs derived from the GBS approach have been reported in important crop species such as barley [5,6], wheat [5], rice [7], raspberry [8] and cotton [9]. In non model crops, new technologies as GBS have not been widely used so far. However in cassava, one of the most highly dense genetic maps was created using GBS-based SNPs, for mapping the resistance to cassava mosaic geminiviruses [10].

Cassava (*Manihot esculenta* Crantz) belongs to the Euphorbiaceae family, which includes approximately 6,300 species [11]. Botanically it is a tropical perennial shrub whose origin center is the Amazon Basin [12]. Cassava typically is a diploid species (2n = 36) [13,14] highly heterozygous and vegetative propagation through stakes in agriculture. Cassava is important for food security in tropical regions of the world. It represents an important source for calories for more than one billion of people [15]. The species tolerates drought and has been considered as a well adapted crop facing climate change which could position it as one of the best alternatives for providing food for the rapidly growing world population in future [16-18].

Cassava is cultivated in more than 100 countries and its leaves and roots can be consumed as food and feed [19]. The plant has also important industrial uses, mainly for its low-cost starch which finds a diverse range of applications [17,20]. For many decades the use of cassava was limited to subsistence of farmers, but since several years is becoming increasingly important for agro-processing industries mainly due to its biofuel potential [21]. Despite the fact that cassava is one of the major crops in the world, a decade ago this crop was listed as one of the least studied plant species [22]. The employment of modern molecular tools will help to go deeper in the understanding of the genetic basis and even lead to the identification and cloning of genes controlling agro-economic importance traits. Most of the genes characterized so far in model and cultivated plants have been cloned employing map based cloning approach [23-26]. The application of this strategy requires the development of high resolution genetic maps [24,27]. The lack of these maps has hampered so far the cloning of interesting genes in cassava [28-37].

While in genetic maps, markers, genes or loci are ordered based on recombination frequencies at meiosis [38], physical maps present ordered fragments of cloned genomic DNA fragments and whose sizes and distances are given in base pairs (bp). Genetic maps have considerable relevance for the construction of comprehensive physical maps. Combining the relative location and order of genetic markers on a map, with their location on scaffolds or contigs allows the assembly of these fragments into a genome-wide physical map [39].

The current draft of the cassava genome sequence (draft v4.1) is publicly available at the JGI’s Phytozome v10 platform and it was obtained by a whole genome shotgun (WGS) strategy [40], using 454 Life Sciences technology. The cassava genome assembled into 12,977 scaffolds span a total of 532.5 Mb [41]. However, based on nuclear DNA quantity, it has been estimated that the cassava genome to be 772 Mb [42]. Strategies based on correlations between physical and genetic maps could serve as one valuable tool for subsequent identification of genes involved in interesting traits [43,44], for genome organization studies [45], assessment of genetic diversity [46] and comparative genome analysis [47].

One the main advantages of genetic and physical mapping is the possibility to integrate traits of interest and the corresponding function of genes [36,48,49]. The availability of the functional maps is of importance not only to better understand the evolution of plant species through synteny but also for marker-assisted breeding programs.

Cassava, like other crops is affected by pests and diseases caused by bacteria, viruses, fungi, phytoplasms and oomycetes [18]. The molecular analysis of plant pathogen interactions in several model plants and crops has allowed the identification of two main branches in plant immunity depending on the receptor molecules involved [50]. One branch is defined on the presence of pattern recognition receptors (PRRs) that are able to detect microbe-associated molecular patterns (MAMPs) [51]. The PRRs have conserved domains as for example leucine rich repeats (LRR), LysM and kinases [52]. The MAMP-triggered immunity (MTI) is effective against non-adapted or non-host pathogens. Some pathogens adapted to infect and colonize particular plants species, suppressing the plant MTI by delivering effector proteins into the plant cytoplasm [53]. However, plants evolved resistance (R) proteins, which recognize specifically some of these effectors and trigger the second branch of immunity named race specific, gene for gene resistance, or effector triggered immunity (ETI) [54]. The largest class of R proteins contains NB-ARC (Nucleotide-binding domain shared by Apaf-1, R gene products, and CED-4) and LRR domains which can be accompanied by the presence of a TIR (Toll/interleukin-1 receptor) domain in their N-terminus. [23,55,56]. Several studies have employed the presence of these conserved domains to identify *R* genes in plant genomes to gain insight about their genome organization and evolution [56,57]. The genome-wide identification of a set of classical defense-encoding sequences and their localization in a genetic map will provide insights into the diversity of genes coding for immunity-related proteins (IRPs) available in cassava and also can contribute to accelerating the process of isolation and cloning of *PRR* and/or *R* genes.

In the present study a new genetic map of cassava is constructed based on a population of 132 F1 full-sib progeny derived from a biparental cross and SNP markers obtained using the GBS approach. Physical mapping of scaffolds from cassava whole genome sequencing using the mapped markers as anchors is presented. Furthermore we present a genome-comprehensive repertoire of cassava IRPs based on the presence of conserved domains. Finally, more than five hundred of genes encoding for IRPs were unambiguously localized on the sequencing scaffolds and on the genetic map.

## Results

### Genotyping by sequencing

To identify polymorphisms the parents and the progeny of the mapping population were genotyped using the GBS approach. On average 2,920,870 reads were generated for each of 134 samples and 2,173,235 tags were obtained in total. Considering that the average length of each tag was 64 bp, the total amount of DNA sequence analyzed was 139 million base pairs. To eliminate possible false positive SNPs, only tags aligned to unique positions in the cassava reference genome were selected. After the alignment to the cassava genome [41], 1,185,928 tags (54.6%) were aligned to unique positions while 229,629 tags (10.6%) were aligned to multiple positions and the remaining 757,678 tags (34.9%) could not be aligned.

In total, 78,854 SNP markers were obtained which corresponds, on average, to one SNP every 1,763 base pairs. They are distributed across 3,450 scaffolds from 12,977 constituting the current cassava genome sequence draft, corresponding to 87% (463.2 Mb) of the genome. The distribution of tagged scaffolds, the number of SNPs representing the scaffolds and the cumulative scaffold length in base pair across the genome is shown in Additional file 1.

From the resulting set of 78,854 SNPs, 51.4% (40,561) of the total set of SNPs correspond to transitions and 48.6% (38,293) to transversions, for a transition-transversion ratio of 1.06. A meaningful number of SNPs, 62.6% (49,429), were located in annotated cassava genome regions. Of these, 52.6% (26,030) were found within annotated CDS (Coding DNA regions). For non-coding regions, 31.7% (15,708) were found within introns, 10% (4,940) within promoters and 5.5% (2,751) within UTRs (Additional file 2).

The gene ontology (GO) analysis was performed for 14,384 unique cassava genome annotated sequences that contain at least one of the 49,429 annotated SNPs obtained by GBS. On average, each annotated region contains three SNPs. In total for the three groups, 2,682 unigenes (counts for gene product characteristics) were obtained corresponding to the 49,429 annotated SNPs. The functional group with the highest gene product counts was biological process with 58.2% (1,562 tags) followed by molecular function 30.2% (811 tags) and cellular component 11.6% (309 tags) (Additional file 3).

### High-density genetic map construction

The obtained 78,854 SNPs were subjected to a series of selective criteria in order to choose the useful SNPs for the purpose of genetic mapping. From the total set of markers, 43,921 SNPs (55.6%) correspond to polymorphic markers in the two parents, from which 25,968 (59.1%) correspond to genotypes derived from a cross between heterozygous and homozygous parents. Monomorphic homogeneous (both parents having the same allele) markers as well as those with missing data in more than 10% of the population individuals were excluded. After the quality control filters the number of useful and informative loci for mapping was reduced to 7,146. More heterozygous markers were identified in the female parental than in the male. Of the 7,146 markers, 2,528 (35.4%) were heterozygous only in the male parent while 2,158 (30.2%) were heterozygous only in the female and 2,460 (34.4%) were heterozygous for both parents. After the filtering of identical segregation and distortion for linkage analysis and map construction 5,300 SNPs were taken into account to be analyzed using Joinmap 4.1. From them, the software integrated, unambiguously, 2,141 SNP markers onto the newly constructed genetic map. These were distributed in 18 linkage groups, which corresponds to the number of haploid cassava chromosomes (2n = 36; n = 18) [13,14]. The numbering was done according to previous studies (see below). The pairwise recombination fractions and LOD scores obtained using R/qtl indicate strong linkage for all pairs of markers on each of the 18 LGs (Additional file 4).

**Figure 1:**
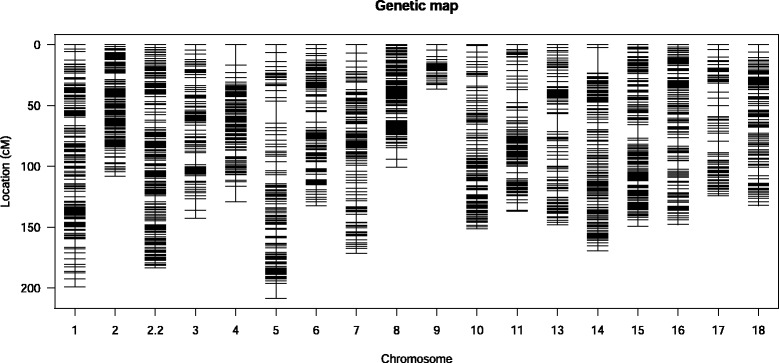
**Cassava genetic map containing 2,141 markers.** The linkage groups are named LG1 to LG18. On each linkage group, the black lines represent mapped markers. Genetic distances are given in Kosambi map units in centi-Morgans and are calculated using JoinMap 4.1 software [86].

The number of SNPs in each linkage group ranged from 35 to 176, with an average of 118.9. The map spanned a total of 2,571 cM, with an average distance of 1.26 cM between markers (Figure 1 and Table 1). The LG5 was the largest group, with a total length of 208.5 cM, while the smallest was LG9, with 36.48 cM. The LG2 and LG8 were the groups with the highest marker density, with an interval of 0.7 cM, whereas the LG17 was the least saturated group, with an interval of 2 cM. Longer intervals were present in linkage groups 5, 4 and 14, with values of 20.7, 18 and 16.6 cM respectively (Table 1 and Additional file 5).

**Table 1:** **Genetic map data summary**

**Linkage group**	**No. of markers**	**Total length (cM)**	**Density Interval (cM)**	**Largest interval (cM)**
1	169	199.09	1.19	7.25
2	156	108.24	0.7	5.42
2.2	176	183.51	1.05	5.06
3	80	142.84	1.81	9.85
4	117	129.03	1.11	16.6
5	120	208.47	1.75	18.03
6	106	132.22	1.26	8.62
7	123	171.58	1.41	6.95
8	146	100.64	0.69	9.32
9	35	36.47	1.07	5.27
10	118	151.38	1.29	8.35
11	113	137.08	1.22	10.87
13	87	148.08	1.61	5.52
14	137	169.35	1.25	20.7
15	154	149.15	0.97	11.11
16	136	147.8	1.09	7.11
17	63	124.07	2	9.23
18	105	13,217	1.27	6.87
Total	2,141	2,571	1.26	

The linkage groups, loci number, total length per group, average distance between markers (density) and scaffolds for each linkage group are shown.

**Figure 2:**
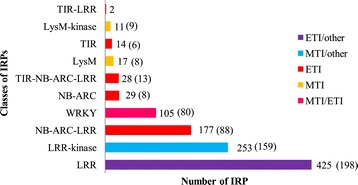
**Repertoire of genes coding for immune related proteins (IRPs) identified in the cassava genome.** Numbers on right of bars show the number for each class of immune related protein. Numbers in parenthesis show the mapped IRPs. The branches of IRPs are indicated by the color code as shown on the upper right side.

**Figure 3:**
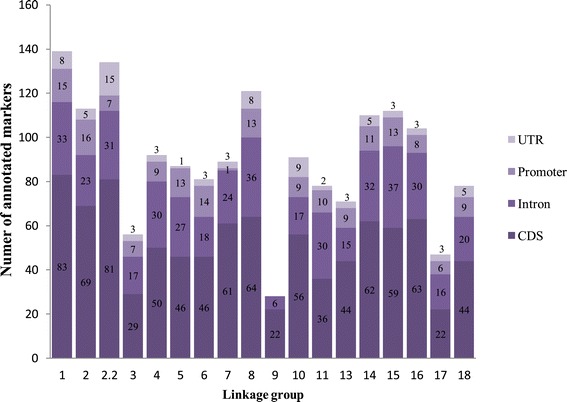
**Summary of mapped annotated SNPs.** Linkage groups and the corresponding annotated loci numbers. The positions of analyzed SNPs in the gene structure are shown by different colors. CDS (Coding DNA Sequence), introns, promoters or UTR (Un-translated Region).

From the total of 2,141 mapped SNPs, 54.6% correspond to transitions and the remaining 45.4% to transversions. 76.1% or 1,631 markers are located in annotated regions, 57.4% (937) are within annotated CDS, 10.4% (170) within promoters, 27% (442) within introns, and 5% (82) within UTRs regions (Figure 2). The total number of annotated markers in the linkage groups varied from 28 annotated SNPs for the LG9 to 139 SNPs for the LG1, with an average of 90.61 SNPs. The LG1 has the highest number of SNPs positioned in CDS regions, followed by LG2.2, while LG9 has the lowest number. For SNPs positioned within intronic regions, the linkage group that has the highest number of counts corresponds to LG15, whereas LG9 again has the lowest number. On the other hand, SNPs positioned in promoter regions, the LG2 shows the highest number of counts while LG9 does not have any. Finally, for SNPs positioned within UTR regions, the LG2.2, LG10 and LG1 have the highest counts (Figure 3).

### Comparative genetic map of cassava

**Figure 4:**
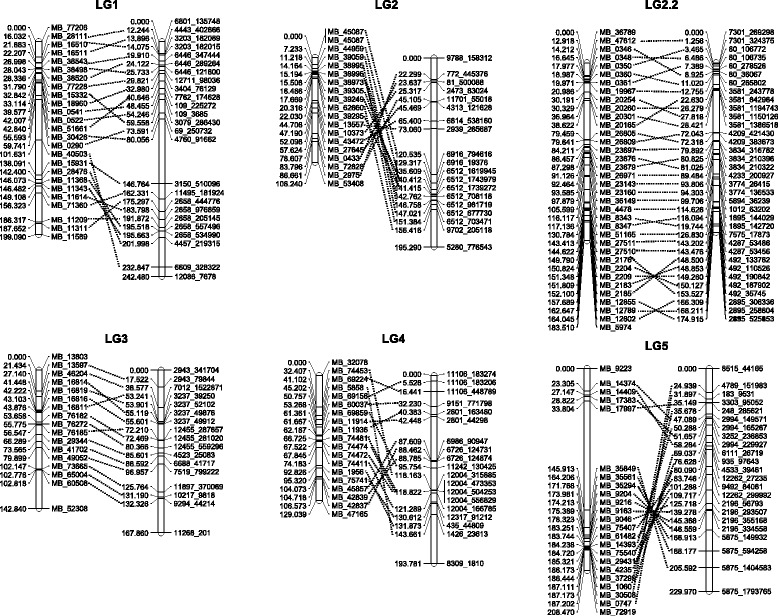
**Anchor markers showing co-linearity between different cassava genetic maps.** Markers with the same genomic position (determined by the corresponding scaffolds) are connected by lines. Comparison was carried out employing the genetic map reported by Rabbi et al [10].

The map constructed here was compared to the previously reported genetic maps [10,58]. Only for the reported LG12 no homologous linkage group could be identified. The rest of linkage groups show high co-linearity when the markers are compared according to the corresponding scaffoldings they tag. The identities of the scaffolds shared for each LG among the maps was in the range between 52% (LG4) and 83% (LG13) with an overall average of 66% throughout all the linkage groups (Figure 4 and Additional file 6). In total 389 anchor markers between the maps were identified. The LG2.2 and LG14 contain the highest anchor markers (34), while the LG17 with 8 markers was the lowest. On average each LG have 21.6 anchor markers (Table 2 and Additional file 6). An additional comparative analysis was done with the cassava map developed by Rabbi et al. [58]. Eight anchor markers distributed in LG1, LG6, LG14, LG16 and LG19 were identified (Additional file 6).

**Table 2:** **Comparative analysis of cassava physical maps**

**LG**	**Nr. of scaf. (A)**	**Nr. of scaf. (B)**	**Common scaffolds**	**Nr. of new scaffolds (B)**	**Size of new scaffolds (bp)**	**Anchor markers**
1	65	45	33	8	885,261	23
2	50	43	25	18	3,391,767	17
2.2	58	43	30	11	1,428,130	34
3	56	35	25	10	1,066,798	15
4	56	33	21	10	813,846	17
5	60	48	28	17	4,888,465	21
6	72	42	28	14	1,766,509	21
7	66	34	25	8	3,061,711	23
8	79	53	37	15	2,262,879	23
9	19	10	10	0	0	16
10	54	37	24	11	1,608,816	23
11	41	32	13	16	2,783,942	11
13	60	32	25	6	1,124,354	16
14	91	46	39	6	329,758	34
15	50	36	25	11	1,026,082	31
16	64	46	31	12	1,996,766	25
17	71	28	22	5	890,465	8
18	60	44	32	11	1,396,841	31
**total**	**1,072**	**687**	**473**	**189**	**30,722,390**	**389**

Unique scaffolds in the reported map version (A, Rabbi et al. [10]), in the map from the present study (B), common scaffolds between them, new mapped scaffolds from this study (B) anchored, their size in bp and the anchor markers per linkage group.

### Physical mapping of scaffolds in the genetic map

To orient the scaffolds of the cassava genome draft sequence into the genetic map, the mapped markers were employed as anchors. A total of 687 unique scaffolds were localized on the genetic map, representing 45.6% (242.6 Mb) of the current cassava reference genome. The linkage groups with the highest number of scaffolds were LG8 (53), LG5 (48), LG16 and LG14 with 46 each. LG9 and LG17 have the lowest numbers of scaffolds with 10 and 28, respectively (Table 2). A total of 46% (316) of the selected scaffolds were tagged by single-markers, 41% (282) were tagged by 2-5 SNPs and 13% (89) by more than five markers. Scaffold 1,551 has the highest count of markers with 45 SNPs in LG15. Only 3.4% (24) of the scaffolds were present in two different linkage groups (Additional file 5). In this way, the previously reported map [58] could be enriched with 189 new scaffolds which were mapped in this study. These scaffolds are disturbed on 17 LGs and the number varied between six for LG13 or LG14 and 18 for LG2. Only for LG9 could not be anchored new scaffolds. In total, the physical map of cassava is extended with 30.7 Mb (Table 2), which correspond to the sum of all new anchored scaffolds.

The relationship between physical and genetic distances in cassava genome was determined. For that, three representative regions were selected from different areas of the LG, one from the middle part and one for each of the distal parts. The scaffolds analyzed contain at least three SNPs. The overall physical map anchored analyzed comprises 32.1 Mb that corresponds to a genetic distance of 215 cM giving a mean value of 603.2 kbp per 1 cM. However, this ratio varies strongly between the linkage groups, from 76.8 to 2,429 kbp per 1 cM in LG13 and LG18, respectively. This variability is calculated also inside of the linkage groups indicating uneven recombination events. In LG11, 1 cM can correspond to 0.1 or to 2,395kbp, whereas in LG2 it ranges from 288.6 to 1,148kbp (Table 3).

**Table 3:** **Relationships between genetic and physical maps, representative for each linkage group and for the whole genome**

**Linkage group**	**Physical length analyzed (kbp)**	**Genetic length analyzed (cM)**	**Mean value of relationship of genetic (1 cM) to physical (kbp) length**	**Range of relationship of genetic (1 cM) to physical (kbp) length**
1	2,550	26.4	169.1	95.2 – 269.3
2	2,048	5.3	751.6	288.6- 1,148
2.2	991	9.37	98.1	83.6 – 125
3	1,780	12.4	144.8	21.6 – 234.7
4	1,810	4.91	1,554	3.5 – 5,273.3
5	1,547	16	167.1	42.3 – 245.8
6	796	3.8	288	18.1 – 680.2
7	1,908	18.2	1,062	32.3 – 3,052.8
8	2,516	8.3	323	62.5 – 562.7
9	577	8.1	92.1	28.9 – 208.1
10	2,065	17.1	332	5.2 – 940.6
11	3,13	4.5	913	0.1 – 2,395
13	2,175	22.8	76.8	7.7 – 209.3
14	2,293	7.9	400	72.6 – 570.2
15	4,633	11	1,561	18.9 – 4,634.6
16	1,759	17.8	296	64.2 – 665.4
17	1,289	6.6	201	82.8 – 420.1
18	1,333	14.1	2,429	8.3 – 699.8
Genome-wide	32,1 Mb	215	603.2	0.1 – 5,273

### Repertoire of immunity-related proteins

Employing a bioinformatics approach, the cassava proteome was investigated for proteins containing the conserved domains present in PRRs and R proteins. A repertoire of proteins with a complex pattern of combinations of these conserved domains was obtained (Figure 2). In total 1,061 IRPs were identified (Additional file 7). From them, 253 were classified as LRR-kinases based on the presence of leucine-rich-repeat and kinase specific domains. These proteins, also known as receptor-like kinases (RLKs), which contain an extracellular LRR and a cytoplasmatic kinase domain are involved in MTI pathways. Seventeen putative proteins containing only the LysM domain and eleven proteins containing both the LysM and kinase domains were detected (Figure 2).

The cassava proteome contains 28 TIR-NB-ARC-LRR, 177 non-TIR-NB-ARC-LRR putative proteins, and two with TIR-LRR domains. Proteins containing only the NB-ARC domain or only the TIR domain were relatively well represented, with 29 and 14, respectively. Proteins with an extracellular LRR domain are also known as receptor like proteins (RLPs) can participate as immune receptors, while other RLPs participate in plant development. The cassava proteome contains 425 of these RLPs proteins. Although the WRKY domain separately is not present in any known R protein, it is present in an important family of plant transcription factors related with defense against pathogens. The cassava proteome has 105 WRKY proteins and none of them contains additional conserved domains (Figure 2 and Additional file 7).

### Genomic organization of immunity related annotated genes

In total, 554 scaffolds containing genes coding for IRPs were identified. Most of the genes, 713 (67%) were localized in scaffolds containing two or more IRPs. However 349 genes (33%) were localized in scaffolds as single genes. The scaffolds containing the highest number of annotated genes encoding for IRPs were 8265 with 13 (5 LRR, 4 LRR-kinase, 3 NB-ARC-LRR and 1 WRKY) and 05875 with 12 (4 LRR, 4 LRR-kinase, 2 NB-ARC-LRR, 1 LysM-kinase and 1 NB-ARC). Scaffold 8,686 contains 11 genes all from the LRR class. Three scaffolds contained ten genes: 6,914 (4 NB-ARC-LRR, 3 LRR, 2 LRR-Kinase, 1 WRKY), 7,520 (5 LRR, 3 LRR-kinase, 1 NB-ARC-LRR, and 1 WRKY) and 10,217 (6 NB-ARC-LRR and 4 LRR). Interestingly, from the 28 annotated genes coding for putative TIR-NB-ARC-LRR proteins, 10 were grouped into only two scaffolds, one containing six genes (scaffold 97) and the other one (scaffold 11,897) containing four of these genes. The six genes in scaffold 97 are located in a region of just 77,359 bp, whereas the four genes in scaffold 11,897 cover 116,966 bp. Scaffolds 3,921 and 11,106 also harbor a relatively high number of genes of the NB-ARC-LRR class, with six genes each. The scaffolds containing genes coding for proteins with a WRKY domain harbor only one or two of this class of genes and only a few have three (Additional file 7).

**Figure 5:**
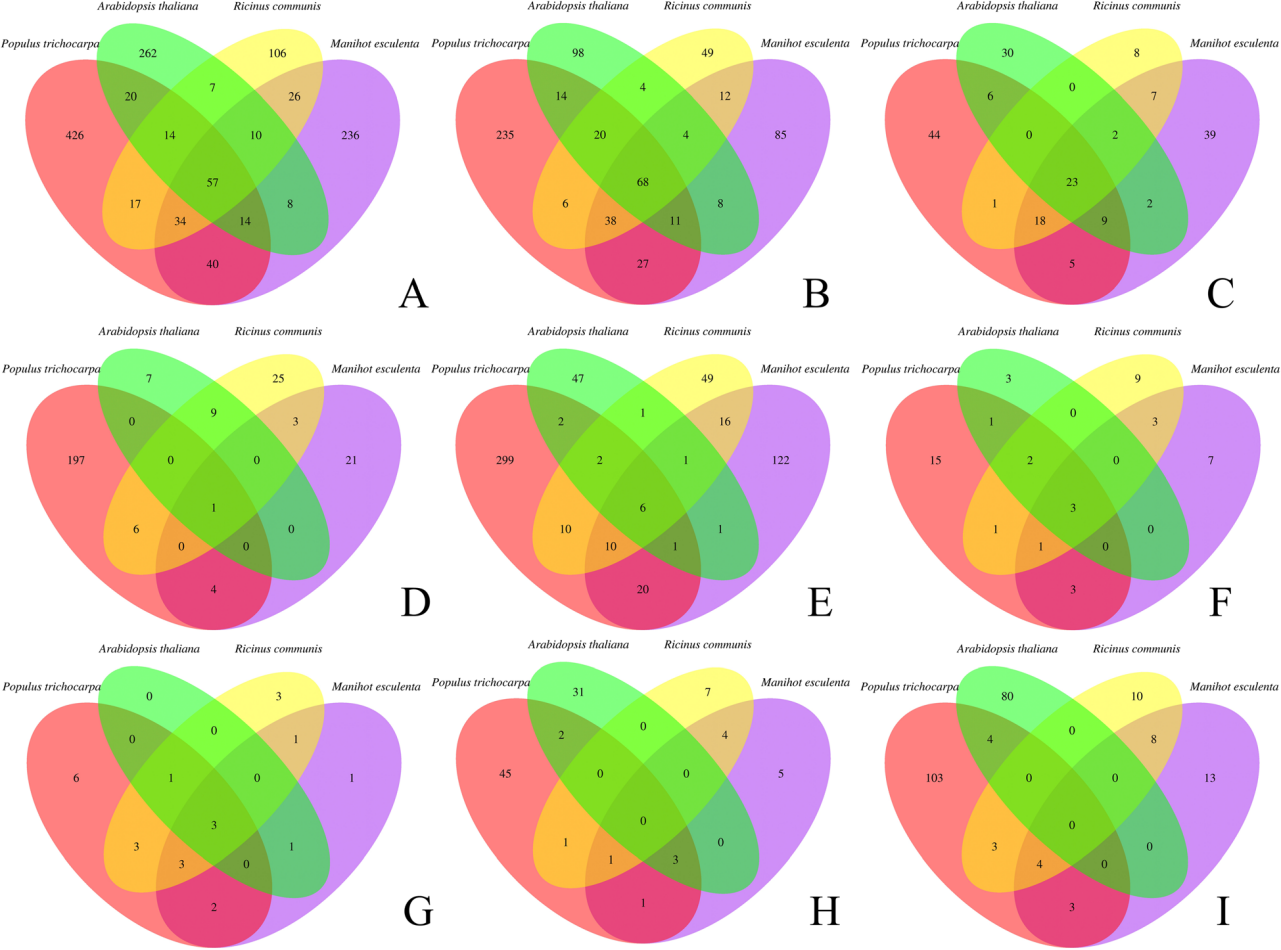
**Orthology clusters between of the predicted immunity-related proteins in** ***Manihot esculentac Arabidopsis thaliana*** **,** ***Ricinus communis*** **,** ***Populus trichocarpa*** **.** **A**. LRR. **B**. LRR-kinase. **C**. WRKY. **D**. NB-ARC. **E**. NB-ARC-LRR. **F**. LysM. **G**. LysM-kinase. **H**. TIR. **I**. TIR-NB-ARC-LRR.

The annotation of the immunity genes in the cassava genome was performed with an Ortholog Cluster Analysis (sequence homology) (Figure 5). *Arabidopsis thaliana*, *Ricinus communis*, and *Populus trichocarpa* were selected as related species and the same pipeline employed to identify conserved domains in cassava was applied for these species. From the 425 putative proteins of cassava classified as LRR proteins by HMMscan, 189 have orthologs with LRR proteins from at least one of the other species analyzed (Figure 5A). A cluster with 57 LRR family proteins was shared by all the three species. Cassava shares 40 orthologous LRR proteins with *P. trichocarpa,* 26 with *R. communis,* and eight with *A. thaliana* (Figure 5A). The second biggest group was the LRR-kinase family. Of the 253 proteins LRR-kinase proteins predicted in cassava, 168 had an orthologous at least in one of the other plant species analyzed. There were 68 orthologs of LRR-kinases shared by all species (Figure 5B). Of the 105 WRKY proteins from cassava, 66 have an ortholog in at least one of the other plant species analyzed and 23 are in a cluster in all species (Figure 5C). In the case of the NB-ARC family, all the 29 cassava predicted proteins had an ortholog in at least one other plant species evaluated and one protein is shared by all of the species (Figure 5D). Of the 177 proteins predicted in the non-TIR-NB-ARC-LRR family, 55 cassava proteins had an ortholog in at least one other analyzed species and six proteins had orthologs in all the studied species (Figure 5E). Finally, less than 15 orthologs are found among the analyzed species for the predicted ORFs of each of the following classes: LysM, LysM-kinase, TIR and TIR-NB-ARC-LRR (Figure 5F-I).

### Mapping of immunity related proteins

Based on the cassava IRP repertoire (1,061 in total), those located on scaffolds oriented in the physical map were selected. In total, 569 IRPs were mapped, 198 of them (34.7%) belonging to LRR class, 1609 (28.1%) to the LRR-kinase, 88 (15.4%) to NB-ARC-LRR, 80 (14%) to WRKY, 8 (1.4%) to NB-ARC, 13 (2.3%) to TIR-NB-ARC-LRR, 8 (1.4%) to LysM, 6 (1.1%) to TIR and 9 (1.6%) to LysM-kinase (Figure 2, Additional file 7).

**Figure 6:**
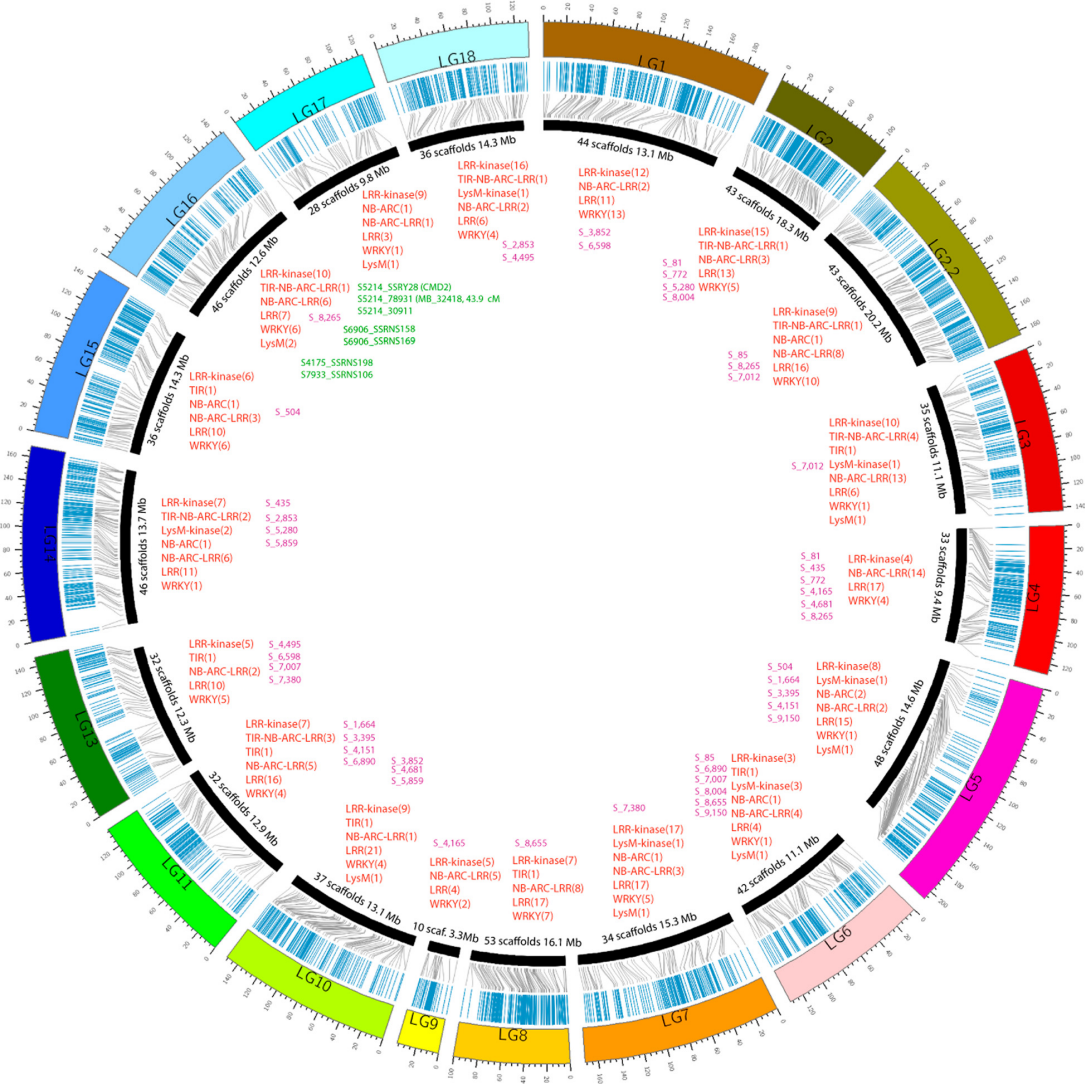
**The cassava genetic and physical map enriched with duplicated scaffolds, IRPs and QTLs for cassava disease resistance.** The linkage groups are highlighted with different colors and the markers in blue lines. In the inner part the black curves mark the anchored scaffolds, their number and cumulative length in Mb per linkage group, orientation based on map positions of markers. In red are shown the IRPs families, their number per linkage group is shown in parenthesis. In purple the duplicated scaffolds and in green the reported loci and QTLs for cassava mosaic virus resistance. The grey lines mark the link between genetic and physical scaffold positions of marker clusters in the same scaffold. Diagram was plotted using Circos software [89].

These 569 genes coding for IRPs were physically located in 226 scaffolds and distributed in all the 18 linkage groups with an average of 31.6 per linkage group. LG2.2, LG7 and LG8 had the highest counts with 45, 45 and 40 genes, respectively. The linkage groups with the lowest counts were LG17 and LG9 with 16 genes each (Additional file 7). In total, 128 clusters were identified, with 382 genes, counting for almost 67% of the total mapped IRPs. Clusters were found in all 18 linkage groups. The cluster with highest number had 11 IRPs (LRR) and was located in LG10, followed by LG3, LG7 and LG18 with clusters of 9 IRPs each. Seventy clusters, on 17 LGs, except for LG13, have two IRPs each. These clusters had diverse combinations of IRP classes (Figure 6).

### Anchoring previous QTLs for disease resistance

We searched to localize loci or QTLs previously reported in our genetic or physical map. The markers SSRY28 (CMD2), S5214_78931 and S5214_30911 have been genetically associated with CMD resistance [10,58-61]. These markers were anchored in the scaffold 5,214 in LG16 (Figure 6 and Additional file 7) at the same position as reported by Rabbi et al. [10]. In this study it was possible to anchor the markers SSRNS158 and SSRNS169 previously associated with CMD resistance [62] in the scaffold 6,906, while in the scaffolds 4,175 and 7,933, localized in the LG16, were anchored the markers SSRNS198 and SSRY106 where a QTL for CMD resistance have been reported [60,61]. Interestingly, from these scaffolds, the 5,214 and 4,175, one (from the LysM family) and five genes (two LRR-kinase, two NB-ARC-LRR and one LysM) coding for IRPs are present (Additional file 7). A fine mapping and/or association studies will allow if these candidate genes are directly related to CMD resistance.

## Discussion

In this work a GBS approach was carried out to identify SNP derived markers in a cassava population for genetic and physical mapping purposes. The 78,854 GBS-SNPs obtained cover 87% (463.2 Mb) of the current cassava genome sequence. These markers were distributed homogenously through 3,450 scaffolds of the genome sequence draft. These scaffolds cover the majority of the cassava genome, although they represent 16.5% of the total number of genome scaffolds. This due to just 487 of almost 13,000 scaffolds covers half of the current cassava genome [41]. No SNPs were identified in small scaffolds representing the remaining 13% of the cassava genome. Consequently, these data constitute the most representative genotyping information for a cassava population until now, and can be relevant for future applications where DNA fingerprint is pivotal.

The transition-transvertion ratio of the total of SNPs was 1.06. This figure is lower when compared to previous cassava reports on genome-wide polymorphic discovery (1.24) [14] and expressed sequence tags (EST) (1.27) [63]. More than 60% of the SNP markers obtained were located within annotated and coding regions. The enzyme *Ape*KI used for preparation of GBS libraries is partially methylation sensitive [2], and this leads to the preferential restriction of coding sequences. Similar results were obtained in cattle using the enzyme *Pst*I, also a methylation sensitive enzyme [64]. SNPs located more often in cassava CDS than in UTRs, which has also been reported in a previous study based on genome-wide analysis [14]. Those SNPs located within a CDS can potentially modify the encoding amino acid chain, resulting in proteins with new functions or introduction of a stop codon. These represent an outstanding source of information to validate the function of genes [65,66] and constitute a direct and effective way to conduct phenotype association analysis.

On the other hand, the SNPs positioned in non-coding regions such as introns might also play key roles in processes of alternative splicing and can be employed in evolution and diversity studies [67]. Those SNPs residing in UTR regions or promoters represent control points to regulate gene transcription and translation. Interestingly, some non-coding regions have been reported as key in regulating and controlling the expression of genes responsible for agronomical important traits such as flowering time in maize [68,69] and loss of seed shattering in rice [70]. Therefore, in this version on the cassava genetic map the description and putative function for the sequences containing SNPs was not limited to coding regions, but to all annotated sequences containing a marker.

The cassava population used in this study is derived from a cross between highly contrasting parents for several phenotypic and phenological traits [22,71]. This cross has been employed so far to identify genomic regions involved in morphological traits [22,28] resistance to CMD (Cassava Mosaic Disease) [59] and Cassava Bacterial Blight [72,73]. The highly dense genetic map reported here could contribute to future research focused on studies of allelic variation and the effect on different traits, as well QTL analysis and marker-assisted breeding programs.

The linkage map we have constructed is the second most saturated map on cassava reported so far [10]. However, although these two maps employed GBS derived markers and the same restriction enzyme for library construction, the total number of SNPs obtained was different. This could be due to library preparation, technical issues, pipeline used for the SNP calling [74], the quality, quantity and concentration of the DNA sample, but also because of the level of genetic diversity between the parents.

The map contained 2,141 SNP markers, distributed in homogenous manner in 18 linkage groups, with a density of 1.26 cM. Some regions of this map are sparsely saturated, as has previously been reported for other species using SNPs obtained from GBS [6,8,10]. This fact could be explained by the scarcity or even lack of polymorphisms in these regions. However, more than 93% of the map shows a high saturation and reduced interval lower than 3 cM. It will be very useful establishing close relationships between markers and QTLs [1,75], facilitating the subsequent identification of genes involved in interesting traits.

Almost half (264.4 Mb) of the current cassava genome draft sequence could be anchored to the genetic map through 687 scaffolds. Comparative map analysis with the reported cassava maps [10] revealed high correlations between linkage groups based on anchor markers. Moreover, the physical map of cassava was extended with 30.7 Mb by anchoring 189 new scaffolds. This will contribute to the efforts of improve the current cassava genome sequence draft. It is expected that SNPs belonging to the same scaffolds to be in clusters on the same linkage groups. Nevertheless, cluster of markers from the same scaffold are disrupted by some markers from other scaffolds. For instance in LG15, scaffold 1,551 was disrupted by scaffold 3,241; in LG2.2, scaffold 2,895 was disrupted by scaffold 4,060. Similar scenarios have also been reported [35-37]. On the other hand, it was found that 24 scaffolds are located at two locations belonging to different linkage groups as already reported by Sraphet et al. [35]. The scaffolds 8,265 and 4,165 seem to harbor duplications, because these two scaffolds are located in more than one LG in the cassava maps [10,35]. Scaffold 8,265 is located in LG2.2, LG4 and LG16 in the map constructed in this study as well as in that reported by Rabbi et al [58]. Scaffold 4,165 is located in LG4 and LG9 in our study but only in LG9 in Rabbi et al [58]. It is common to assume that the genomes of plants of the same species are similar, however, there is increasing evidence for rearrangements, translocations, gains or losses of DNA segments and copy number variations (CNV) usually found in all chromosomes among the genomes of different genotypes of the same species [76,77]. This might be the case between the genotypes used for the draft genome sequence and the parents used in this study and might explain the differences observed between the genetic and physical map found. Undoubtedly, a consensus genetic map for cassava could be helpful in this regard, as has been performed for other species with high heterozygosity level such as grapevine and apple [78,79]. Other explanations might be that some of the markers identifying these scaffolds are not properly mapped or because of errors during assembly of the reads, that are still present in the draft genome sequence.

The relationship between physical and genetic distances found is the range of reported data for other plant species. The value of 603 kbp for 1 cM determined in this study for cassava varies between 139 kbp in Arabidopsis to 510 in tomato or 2140 in maize (http://www.ndsu.edu/pubweb/~mcclean/plsc731/analysis/analysis5.htm). This information is useful when detailed genome structure analysis or gene cloning by map-based cloning approaches will be undertaken in the future.

A high number of SNP-tagged genes were classified in different GO categories, showing a wide variety of functions in the annotated regions containing markers. This represents a meaningful source of genes/markers, which can be employed to answer important biological questions and set up of further experiments to confirm gene functions and links with phenotypes. GO analysis is a basis for construction of functional maps for a particular group of genes of one of the functional categories, such as responses to abiotic or biotic stress. Moreover, it allows the quick mapping of gene families or even gene pathways for interesting traits.

Based on the presence of conserved domains in the PRR and R proteins, it was possible to identify a large IRP repertoire in the cassava genome. In total 1,061 IRPs were identified, although probably not all of them are involved in plant immunity. The next challenge will be to identify the MAMP or effectors that are recognized by these predicted proteins. The numbers of IRPs varies enormously between plant species. For example, the quantity of NB-ARC-LRR, the largest class of R proteins, ranges from 92 in *Brassica rapa* [80] and 150 in *Arabidopsis thaliana* [81] to 438 in potato [57]. The reasons for the number variation of IRPs between different plant species have not been explained so far.

In other plant genomes, more than 40% of genes encoding for IRPs are clustered and the cluster size can be highly variable [57,80,81]. In cassava we found a range from two to eleven members per cluster whereas in *Arabidopsis* was from two to seven [81], or two to eighteen in potato [57]. As the physical map reported here represents 45.6% of the current cassava genome, it is expected that more IRPs and clusters of them lie in the remaining genome regions that could not be analyzed. The 1,061 IRPs were analyzing 532 Mb sequence information. This information will be important to infer the evolutionary history of these important genes and better understand how their genome organization has influenced on their structure dynamics and adaptation to pathogen-derived selective forces.

In addition, in this study it was possible to anchor some markers with scaffolds present in the LG16 with a region containing loci associated with CMD reported previously. This example shown the utility of how dense genetic and physical map information in addition of phenotypic is an excellent way to accelerate the cloning of agronomic interest trait genes or to develop markers useful in marker assisted selection programs. With more phenotypic and QTL analysis the association between the markers identified in this study and traits will increase.

## Conclusions

To our knowledge, this is the first functional map for immunity genes based on an integrative genetic map with anchored sequencing scaffolds from genome draft in cassava. It was possible to anchor almost half of the current cassava genome sequence draft to the genetic map. The map was enriched with 189 new scaffolds that increase the last version of the cassava map in 30.7 Mb. Nearly 344 Mb or 64% of the genome sequence draft is now anchored to the genetic map. On the other hand, the map was also enriched with annotated IRPs and with reported loci associated to cassava mosaic virus resistance. The presented data will allow in the future to map and associate markers with single loci or QTLs for particular traits and molecular cloning of genes controlling these traits. In addition, these data will contribute to future efforts in closing the gaps in the sequence draft and for construction of a cassava consensus genetic map. The cassava IRP repertoire, as well as their genetic and physical map position accompanied with the SNP information will be a reference for future genetic analysis and candidate gene approaches to improve cassava resistance to their diverse biotic diseases.

## Methods

### Mapping population and DNA extraction

The mapping population consists of a full sib F1 segregating population of 132 individuals derived from single seeds of a cross between cultivars TMS30572 and CIAT’s elite cultivar CM2177-2 [28]. Total genomic DNA was extracted from young leaf tissue of 132 individuals of the F1 population and their parents TMS30572 and CM2177-2, using the commercial kit QIAGEN DNeasy Plant Mini Kit® (Hilden, Germany), following the manufacturer’s protocol and adjusting the final concentration to 100 ng/μL. To assess the quality of DNA and absence of enzymatic inhibitors, a restriction digestion was performed using *Hind*III and visualized on a 1% agarose gel.

### Genotyping by sequencing (GBS) approach

GBS libraries were prepared and analyzed at the Institute for Genomic Diversity (IGD, Cornell University, USA), according to Elshire et al. [2]. The partial methylation sensitive *Ape*KI restriction enzyme that recognizes a five base pair sequence (GCWGC) was used for digestion and a library was generated with 134 unique barcodes for progeny and parents. Two lanes of Illumina Hi-seq (Illumina, Inc.) were used for the all samples.

The GBS analysis pipeline 3.0.139 version, an extension to the Java program TASSEL [82], was used to call SNPs from the sequenced GBS libraries [2]. The mean sequencing depth was 8 to 10 times. The alignment of the resulting tags to the reference genome was performed using BWA Version 0.6.2-r126 [83], checking that each SNP has a unique position within the genome scaffolds with 89% of identity. The markers were delivered as Hapmap and VCF (v0.1.10) (Variant Call Format) format files [84].

### Filtering of GBS data

From the complete set of markers an initial filtering was performed using SAS® 9.3 [85] (script, unpublished), to select those SNPs with Mendelian segregation for 1:1 if segregating only in one parent and 1:2:1 if segregating in both parents. Less than 10% of distorted markers were allowed. Monomorphic homogeneous SNPs and those with identical segregation were discarded. The segregation in the population, corresponding to 132 individuals was analyzed for markers that exhibited polymorphisms between TMS30572 and CM2177-2.

### Linkage analysis and map construction

Both linkage analysis and map construction were performed with JoinMap 4.1, and data were analyzed using the CP (outbreedering full-sib family) population type [86]. The *X*^*2*^ test was used to assess goodness-of-fit to the expected 1:1 or 1:2:1 segregation ratio for each marker. Linkage groups were established using a grouping LOD (logarithm base 10 of odds) threshold upper than 3. Markers were assigned to correct linkage groups using two-point grouping analysis and within each group were mapped based on the strongest cross-link (SCL). The map was generated using a recombination frequency below 0.50 and the “ripple” procedure was applied. Recombination frequencies were converted to relative distances in centiMorgans (cM) using Kosambi function [87]. The graphical presentation of the linkage groups was performed using R/qtl [88].

### Comparative genetic map of cassava

The map developed in this study was compared to the other cassava reported maps. For that the SNP markers located at the same position on scaffolds were used as anchors. The genetic positions of these markers were compared and the co-linearity of the maps was determined. The comparison revealed the number of newly mapped scaffolds and their size was determined.

### Physical mapping

All SNP markers obtained were physically localized in the scaffolds of the cassava draft genome sequence (www.phytozome.com), based on minimum sequence similarity of 89%. For that, the core sequence of the marker locus (64 bp) was aligned towards the available genome sequence information to order the position of the markers on the scaffolds. The scaffolds were anchored and the corresponding positions along the cassava chromosomes were defined by comparing the positions of markers on the scaffolds and on the genetic map. The percentage of coverage was calculated as sequence covered by all mapped scaffolds to the estimated total cassava genome size.

The graphical presentation of the physical map was done by using Circos algorithm [89].

### Mapping of immunity-related proteins

The genes taken into account were those encoding for proteins containing any of the following domains or domain-combination: LRR (Leucine-rich repeat), WRKY, LRR-kinase, NB-ARC (Nucleotide Binding domain shared by Apaf-1 R gene products, and CED-4)-LRR, TIR (Toll/interleukin-1 receptor)-NB-ARC-LRR, LysM (Lysin motif)-kinase. All these domains or domain-combination correspond to essential part of the most studied immunity-related protein encoding genes [90,91]. Models for each domain were downloaded from http://pfam.sanger.ac.uk [92]. HMMscan was used with the downloaded models to search the cassava proteome for proteins containing one or more of the selected domains, using an e-value cutoff of 10. Proteins containing several of the domains were identified collapsing the information of the position and presence/absence of each domain. The genomic coordinates of each protein were retrieved using BioMart tool from http://www.phytozome.net/cassava.

In order to detect orthologous clusters in *Manihot esculenta*, *Arabidopsis thaliana*, *Ricinus communis*, and *Populus trichocarpa* the protein prediction using HMMER [93] was performed. *R. communis* and *P. trichicarpa* are chosen as the closest relatives of cassava and *A. thaliana* as model organism for which detailed analysis of IRGs has been reported [81]. The Orthologous Cluster Analysis was done using QuartetS [94]. Two programs, Single Linkage Cluster (SLC) and Markov Cluster Algorithm (MCL) were implemented to cluster genes into orthologous clusters.

Using the obtained catalog of cassava IRPs, the annotated regions containing GBS-markers were identified, to subsequently locate them on the map according to their genome-scaffolds positions. IRP clusters were determined using scaffolds and map positions. The definition of cluster was according to Meyers *et al* [81] and Jupe *et al* [57]. A maximum distance between two or more IRPs of 200 kb was allowed and less than eight non-IRPs between them.

### Availability of supporting data

The SNP data set supporting the results of this article is available in the SNiPlay repository, http://sniplay.cirad.fr/cgi-bin/public_data.cgi. The Cassava draft genome sequence used in this research is available at http://phytozome.jgi.doe.gov/pz/portal.html#!bulk?org=Org_Mesculenta.

## Additional files

Additional file 1:
**Distribution of the genotyping data obtained by GBS across 3,450 scaffolds of the cassava genome.** The table shows the number of SNPs representing the scaffolds in 1,000 range and the cumulative scaffold length in base pairs. Additional file 2:
**Classification of cassava’s SNPs obtained by GBS approach.** The SNPs are classified according to transition or transversion interchanges and by genomic location within an annotated gene (CDS (Coding DNA Sequence), introns, promoters or UTRs (Un-Translated Region). Additional file 3:
**Pie chart of functional categorization of cassava annotated sequences that contain SNPs.** Categorization is based on GO annotation and class sorting based on Plant specific GO slim terms (CateGOrizer tool). A. Biological process. B. Molecular function. C. Cellular component. Additional file 4:
**Plot of pairwise recombination fractions and LOD scores.** The upper left triangle shows the estimated recombination fractions while the lower right triangle shows the LOD scores for all pairs of markers of the 18 LG of the cassava genetic map. The red diagonal indicates strong linked (large LOD values or small recombination fractions). Plot was done using R/qtl [88]. Additional file 5:
**Summary of the genetic map data and anchored genes coding IRPs.** Linkage groups, marker names, cumulative marker distance in cM, scaffold in the draft reference genome, position in bp and corresponding anchored genes coding IRPs start and end position in bp. Additional file 6:
**Detailed comparative analysis of cassava genetic and physical map reported here with those reported previously [** 10,58 **].** Number of unique scaffolds in three map versions, common scaffold number, and new scaffolds anchored, their size in bp and the anchor markers per linkage group, with its respective genetic (cM) and physical (bp) positions in each map. Additional file 7:
**Summary of the immunity related proteins repertoire and its gene sequence position in the cassava genome, scaffold number and start/end position in bp.**
