# Supplementary figures and images for: A genetic map of cassava (Manihot esculenta Crantz) with integrated physical mapping of immunity-related genes
Source: BMC Genomics. 2015 Mar 16;16(1):190. doi: 10.1186/s12864-015-1397-4 (PMC4417308; doi:10.1186/s12864-015-1397-4)

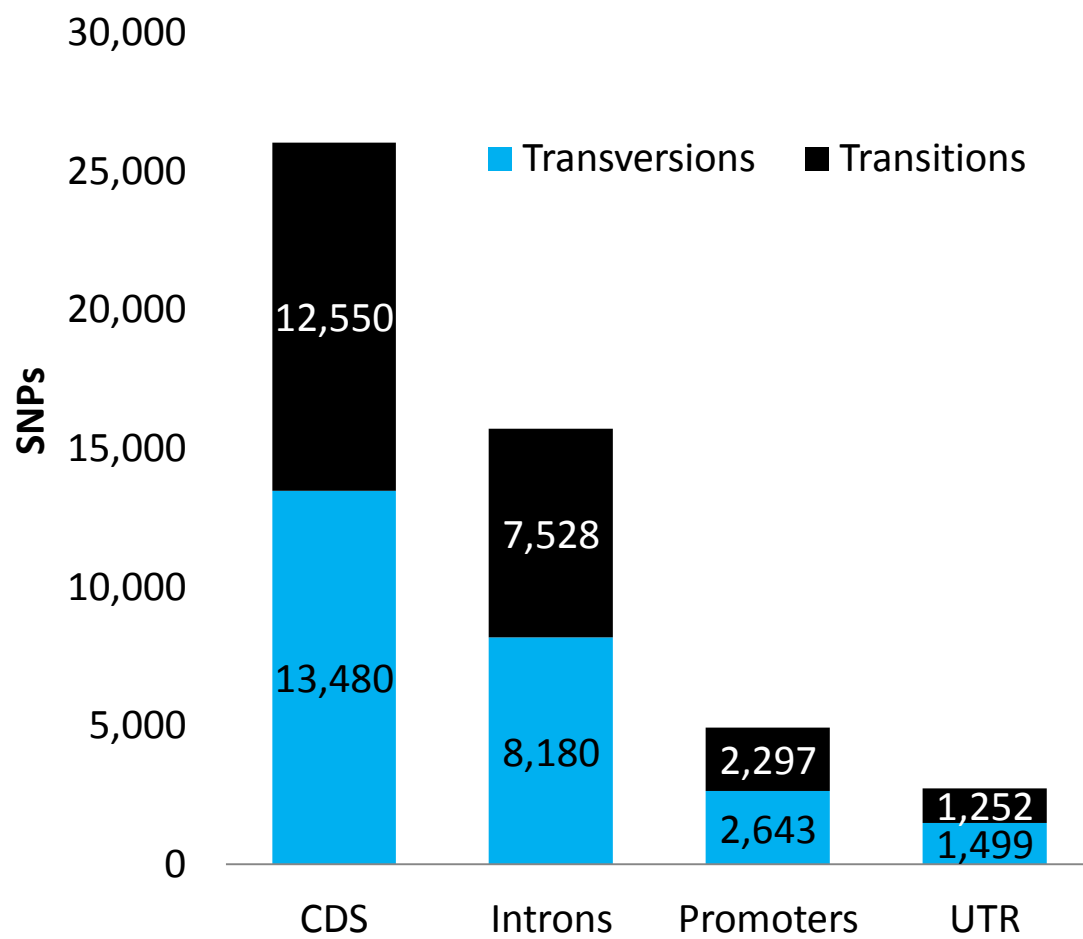

Supplement: Additional file 2: — Classification of cassava’s SNPs obtained by GBS approach. The SNPs are classified according to transition or transversion interchanges and by genomic location within an annotated gene (CDS (Coding DNA Sequence), introns, promoters or UTRs (Un-Translated Region). [file 12864_2015_1397_MOESM2_ESM.pdf]

### a. Biological process

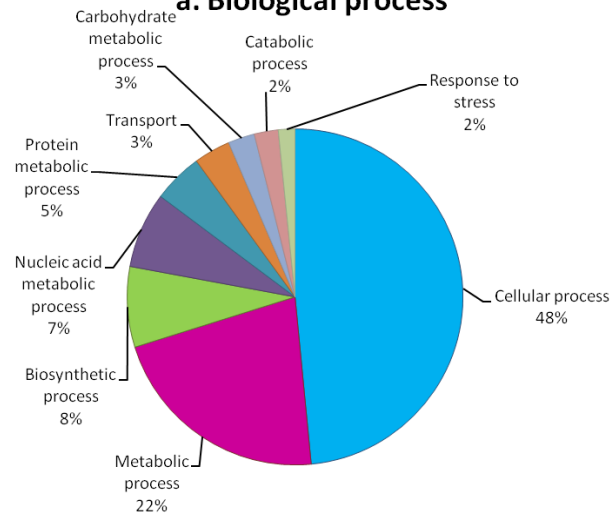

### b. Molecular function

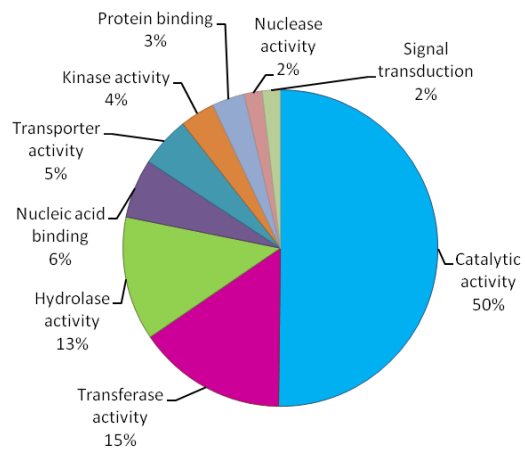

### c. Cellular component

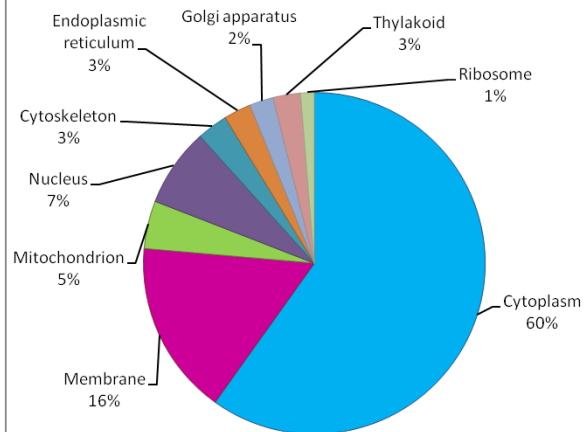

Supplement: Additional file 3: — Pie chart of functional categorization of cassava annotated sequences that contain SNPs. Categorization is based on GO annotation and class sorting based on Plant specific GO slim terms (CateGOrizer tool). A. Biological process. B. Molecular function. C. Cellular component. [file 12864_2015_1397_MOESM3_ESM.pdf]

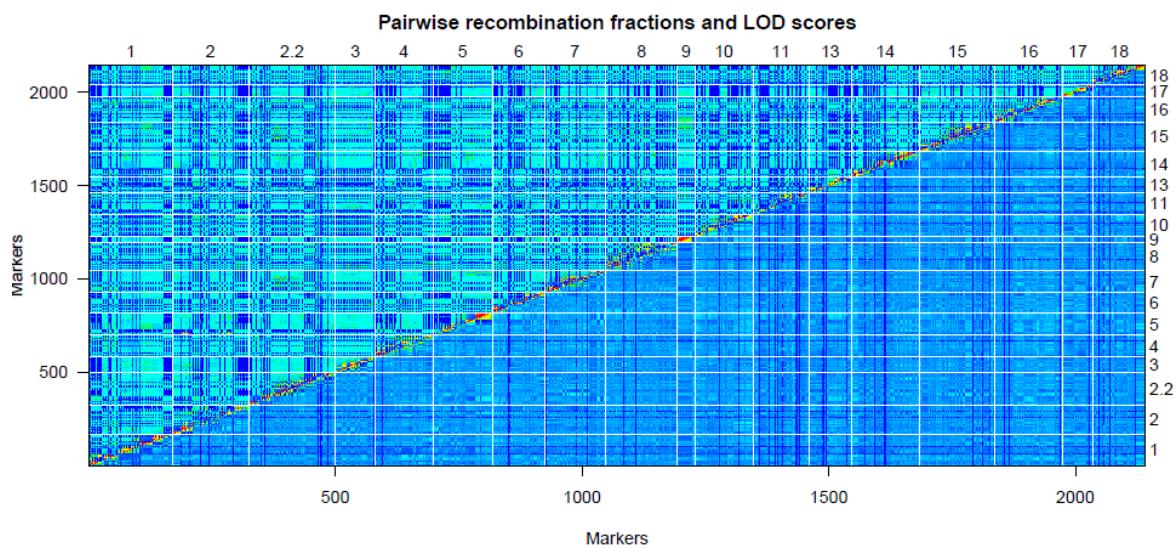

Supplement: Additional file 4: — Plot of pairwise recombination fractions and LOD scores. The upper left triangle shows the estimated recombination fractions while the lower right triangle shows the LOD scores for all pairs of markers of the 18 LG of the cassava genetic map. The red diagonal indicates strong linked (large LOD values or small recombination fractions). Plot was done using R/qtl [88]. [file 12864_2015_1397_MOESM4_ESM.pdf]
